# Non-equilibrium Inertial Separation Array for High-throughput, Large-volume Blood Fractionation

**DOI:** 10.1038/s41598-017-10295-0

**Published:** 2017-08-30

**Authors:** Baris R. Mutlu, Kyle C. Smith, Jon F. Edd, Priyanka Nadar, Mcolisi Dlamini, Ravi Kapur, Mehmet Toner

**Affiliations:** 1 BioMEMS Resource Center, Center for Engineering in Medicine and Surgical Services, Massachusetts General Hospital and Harvard Medical School, Boston, Massachusetts 02114 USA; 2 Massachusetts General Hospital Cancer Center and Harvard Medical School, Boston, Massachusetts 02114 USA; 3 Shriners Hospital for Children, Boston, Massachusetts 02114 USA; 4 MicroMedicine Inc., Watertown, Massachusetts 02472 USA

## Abstract

Microfluidic blood processing is used in a range of applications from cancer therapeutics to infectious disease diagnostics. As these applications are being translated to clinical use, processing larger volumes of blood in shorter timescales with high-reliability and robustness is becoming a pressing need. In this work, we report a scaled, label-free cell separation mechanism called non-equilibrium inertial separation array (NISA). The NISA mechanism consists of an array of islands that exert a passive inertial lift force on proximate cells, thus enabling gentler manipulation of the cells without the need of physical contact. As the cells follow their size-based, deterministic path to their equilibrium positions, a preset fraction of the flow is siphoned to separate the smaller cells from the main flow. The NISA device was used to fractionate 400 mL of whole blood in less than 3 hours, and produce an ultrapure buffy coat (96.6% white blood cell yield, 0.0059% red blood cell carryover) by processing whole blood at 3 mL/min, or ∼300 million cells/second. This device presents a feasible alternative for fractionating blood for transfusion, cellular therapy and blood-based diagnostics, and could significantly improve the sensitivity of rare cell isolation devices by increasing the processed whole blood volume.

## Introduction

Very large volume (hundreds of milliliters) blood fractionation (separation of desired components from whole blood) in a clinically acceptable timescale (a few hours or less) is a formidable challenge with vast implications in healthcare, life science and clinical research. Fractionation is a requirement for generating blood products, as well as an important preparatory step for diagnosis, prognosis and treatment of various diseases. In particular, separation of white blood cells (WBCs) from other blood components is useful for a variety of biomedical applications, including: diagnosis of immune diseases, monitoring of immune response and disease progression, and treatment of leukemic patients (*i.e*. leukopheresis)^1^. Additionally, WBC isolation (*i.e*. buffy coat preparation) facilitates rare cell analysis as buffy coat may contain circulating tumor cells (CTCs), hematopoietic stem cells (HSCs), fetal nucleated red blood cells (NRBCs)^2–4^. Recent studies have shown that these rare cells are critical for developing patient specific therapies, as well as studying associated diseases^5^. Thus, devices that can isolate these rare cells have been developed and are currently being used by both academic and commercial labs^6–8^. Nevertheless, harvesting a sufficiently large number of rare cells (which are typically in the range of 0.1 to 100 cells per mL of blood) remains a challenge due to the limited blood volume that can be processed by these devices. Notably, large volume blood processing would enable CTC-based early cancer detection assays. Therefore, being able to fractionate a large volume of blood in a relatively short time is a crucial precursor to the development of downstream assays for rare cells in blood^9, 10^. In addition to rare cell isolation applications, in the U.S. alone 36,000 units of red blood cells (~300 mL/unit) are needed daily^11^. A key process in the production of red blood cell (RBC) blood products is leukoreduction (*i.e*. reduction of the white blood cell content of the product). Thus, a rapid and scalable blood fractionation method is also highly desirable for transfusion related applications.

Microfluidic devices present a robust, easy to use alternative to conventional, bulk techniques for processing blood (*e.g*. fractionation, separation, sorting) due to minimal need for operator input and intervention. These devices typically use either cell specific markers (via antigen-specific antibodies) coupled with a method to exert force on the cells (*e.g*. magnetic particles/field), or exploit inherent physical differences of various cell types (*e.g*. density, shape, size, deformability). Label-free, size-based cell sorting in microfluidic devices is most commonly achieved using deterministic lateral displacement (DLD), which requires a fine array of posts that directs the cells via physical contact (*i.e*. bumping)^12^. In the first study to use blood in a DLD chip, 20 mL of maternal whole blood was processed in 6 hours to isolate nucleated RBCs^13^. More recently, Ozkumur *et al*. reported a CTC isolation chip that couples a DLD array with magnetic particle separation, and processes whole blood volumes up to 20 mL (8 mL/hour)^14^. Loutherback *et al*. reported a cancer cell isolation chip that ran at a high flowrate (10 mL/min)^15^, but operated only for 30 seconds, processing a diluted sample of 500 μL whole blood. The same research group recently reported that they were able to alleviate the effects of clot formation by using anti-coagulants^16^, and demonstrated processing of 14 mL whole blood volume in 38 minutes (0.37 mL/min). While several other DLD devices have been developed for blood processing applications^9, 17^, they have been limited to processing whole blood volumes up to a few tens of milliliters. Factors that contribute to this limitation include the direct physical interaction between blood components (*e.g*. cells and platelets) and DLD posts, and the requirement for very fine gaps in the array to avoid loss of target cells during bumping, both of which lead to decreased throughput, and potential clogging of the device.

Inertial microfluidics is an alternative method which allows cell manipulation in a gentler fashion, because it does not require contact between cells and the device features. Instead, inertial microfluidics uses lift forces which correlate strongly with the cell size. This method has found significant use in cell focusing (*i.e*. concentrating cells in a single, narrow stream), for applications such as flow cytometry^18^, or facilitating marker-based cell separation^14^. For size-based cell separation, the spiral geometry has been extensively investigated, because it enables Dean flow, which causes the cells to traverse between inner and outer channel walls in a periodic manner. Based on a spiral design, Hou *et al*. reported a CTC isolation device which processes blood at a flowrate of 3 mL/hour, and was able to recover 85% of the CTCs^19^. More recently, Warkiani *et al*. reported a similar CTC isolation device capable of processing 7.5 mL of CTC-spiked lysed blood in 12.5 min (0.6 mL/min), and a comparable CTC recovery rate of 85%^20^. A drawback of the spiral method is the physical interaction of the blood cells with the target cells as the cells traverse in the microchannel, which hampers the overall yield and throughput. Furthermore, the spiral design is difficult to scale in 2D, because of the large footprint (1–10 cm spiral radius) requirement. This issue was addressed by stacking three chips in 3D in the latter study^20^, but it is not an easily scalable procedure as it required physical alignment and plasma bonding of the chips. In a straight channel geometry, Gossett *et al*. showed the transfer of leukocytes from an RBC-free lysate (10x diluted whole blood in red blood cell lysis buffer) to a clean buffer solution where they reached equilibrium, and processed the diluted lysate at a flowrate of 60 μL/min^21^. In summary, current inertial microfluidic approaches are not able to process clinically relevant large blood volumes with the desired high target-cell yield in a short turn-around time.

In this study, we report an inertial microfluidic device called a non-equilibrium inertial separation array (NISA), which can fractionate up to 400 mL of whole blood within 3 hours to produce an ultrapure buffy coat product. This is achieved by processing 1:1 diluted whole blood at approximately 6 mL/min volumetric flow rate (*i.e*. whole blood at 3 mL/min), and at 300 × 10^6^ cells/second. The NISA device is designed as an array of islands, which exert a wall-induced inertial lift force on the proximate cells. Due to the strong correlation between the inertial lift force and cell size, cells follow different size-dependent migration paths towards their inertially focused equilibrium positions. This difference in the cells’ migration trajectories is then exploited for separation by siphoning a portion of the flow from the near-wall region before the cells reach their equilibrium positions. Thus, the smaller cells (*e.g*. RBCs and platelets) are siphoned while the larger nucleated cells (*e.g*. WBCs) are not. Non-equilibrium separation allows significantly shorter island (wall) lengths compared to the required length for inertial focusing, which reduces the pressure requirement of the system, allows for a compact array structure and minimizes the footprint for scalability. Repetitive siphoning in the array provides additional robustness against WBC loss, without causing RBC carryover. We demonstrated that a highly-parallelized NISA device (104 devices fitted on a 12-cm diameter disk) can process blood continuously for up to 3 hours and fractionate 400 mL of whole blood with steady performance (96.6 ± 0.3% WBC yield and 0.0059 ± 0.0006% RBC carryover) over the duration of the operation.

## Results and Discussion

### Non-equilibrium inertial separation array (NISA) mechanism and device design

**Figure 1:**
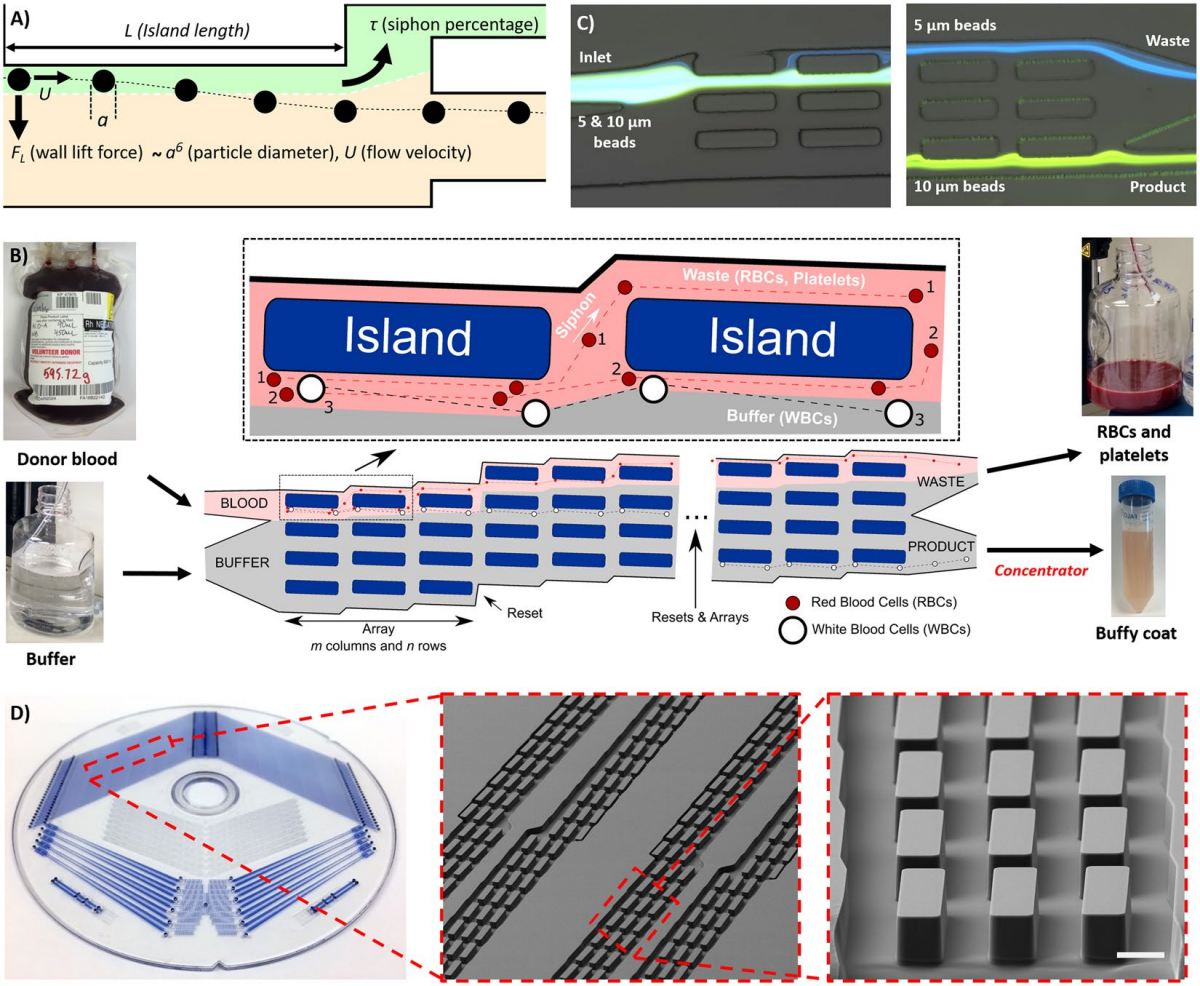
NISA device physics, design and function (**A**) Schematic illustrating the physics of the NISA mechanism. The particle migrates away from the wall due to the wall lift force. After migration, a fraction of the flow is separated (green), but the particle has migrated sufficiently to avoid siphoning. The migration trajectory is dictated by the particle diameter (*a*), flow velocity (*U*), island length (*L*) and siphon percentage (*τ*). (**B**) Schematic of the NISA device utilizing an array of islands and resets. RBCs that start closer to the wall (#1) do not migrate far enough and get siphoned to the waste. RBCs starting farther from the wall (#2) can make it to the next island(s), but eventually get siphoned out. WBCs (#3) can migrate away from siphoning every time regardless of their starting position. (**C**) Inlet and outlet image of the analytical device during separation of 5 and 10 μm fluorescent polystyrene particles to illustrate the mechanism. (**D**) Fully-parallelized NISA device and SEM micrographs showing close-up array structure (Scale-bar is 50 μm).

The physics governing the non-equilibrium inertial separation array (NISA) mechanism, and the overall device design is depicted in Fig. 1A. Briefly, particles flowing in a microchannel experience a wall-induced inertial lift force (*F*_*L*_) that correlates strongly with their size (*a* ^*6*^) (See SI for details)^22, 23^. Thus, different blood components follow different migration paths away from the wall, based on their size. The NISA device is designed to separate the WBCs by siphoning the RBCs (and platelets) that are closer to the wall before they reach their inertially focused positions. The critical design and operation parameters of the NISA device include: island length (*L*), flow velocity (*U*) and siphon fraction (*τ*). Note that the angle of the islands (with respect to the horizontal axis) is not a key factor for separation. Since this study focuses on generation of buffy coat, the siphoned-out portion of the flow (containing RBCs and platelets) is called “waste” and portion with migrated cells (WBCs) is called “product” for brevity.

Based on the NISA mechanism, we developed a microfluidic device to generate a high yield and high purity WBC product from whole blood. This device was designed as an *n* (row*s*) × *m* (*columns*) array of islands, where each consecutive island siphons a fixed portion (*τ*) of blood, until all the blood is siphoned to the waste row (Fig. 1B). Note that the siphon percentage (*τ*) is deterministically set by the device geometry, specifically by adjusting the widths of the waste (top) and product (bottom) rows based on hydraulic resistance calculations. The outcome of this design is that a small portion of RBCs and platelets get siphoned at each island, while virtually all the WBCs migrate away (*i.e*. escape the siphoned fluid) each time. Thus, the WBCs remain in the row in which they begin, while RBCs and platelets are siphoned to the expanding waste row. After 100% of the blood is siphoned (*i.e. m* × *τ* ≥ 100%), a reset is added where all rows move up such that islands are re-introduced to the waste row. This enables WBCs to jump to the row below, one row closer to the product row. Thus, after *n-1* resets it is expected that all the WBCs end up in the product row. By adding extra resets (>*n* − *1*), any WBCs which may have been lost to the waste row can migrate back into the product, which provides robustness against imperfections in device manufacturing and operating conditions. The array design also allows more blood to be processed by the device. While the sample injection fraction (*i.e*. fraction of total flow within the array that is from the sample inlet) for a single island (without a repeating array) would need to be limited to ensure that cells are very close to the wall initially, in an array design, WBCs which do not experience any wall lift force initially will eventually come closer the wall and migrate away as more of the blood is siphoned. A device which was used to visualize this mechanism is shown in Fig. 1C. In the upstream image the smaller (5 μm, blue) particles are siphoned, while the larger (10 μm, green) particles migrate away. After the first island, the width of the stream is slightly reduced, enabling more particles to interact with the wall, which continues until all the stream is siphoned and a reset is added. In the outlet, the small particles remain in the topmost row (waste) while the large particles have migrated to the bottom row (product). The fully-parallelized device, manufactured of cyclic olefin polymer (COP) is shown in Fig. 1D. The three different device designs which was used to investigate, optimize and evaluate the NISA mechanism is summarized in Table 1.

**Table 1:** List of devices used to characterize and evaluate NISA performance.

Name	#Parallel devices	#Arrays/Resets	Per array			Material	Typical processed sample
			#Rows (*n*) × #Columns (*m*)	Row width × Island length × Distance b/w islands × Height [μm]	#Islands		
Single deflection chip	1	—	1	50 (30) × 1200 × N/A × 52	1	PDMS	<1 mL particles/blood cells
Analytical device	4	6	4 × (25 to 42)	50 ^[ii]^×200 × 50 × 52	75 to 126	COP	3 mL 1:1 diluted whole blood
Fully-parallelized device^[i]^	104	6	4 × 34	50 ^[ii]^×200 × 50 × 52	102	COP	800 mL 1:1 diluted whole blood

(I) Single deflection chip was used to characterize the particle/cell migration away from the wall, (II) Analytical device was used to investigate the effects of flowrate and siphon percentage in blood fractionation, (III) Fully-parallelized device was used to verify the scalability and the throughput of the NISA device for very large volume blood fractionation. ^[i]^A concentrator was added at the end of the product row of the fully-parallelized device to reduce the product volume. This concentrator design was reported elsewhere^24^. ^[ii]^50 μm is the fixed width of the middle two rows, where the width of the top and the bottom rows vary to keep the siphon percentage (τ) fixed.

### Characterization of particle and cell migration

**Figure 2:**
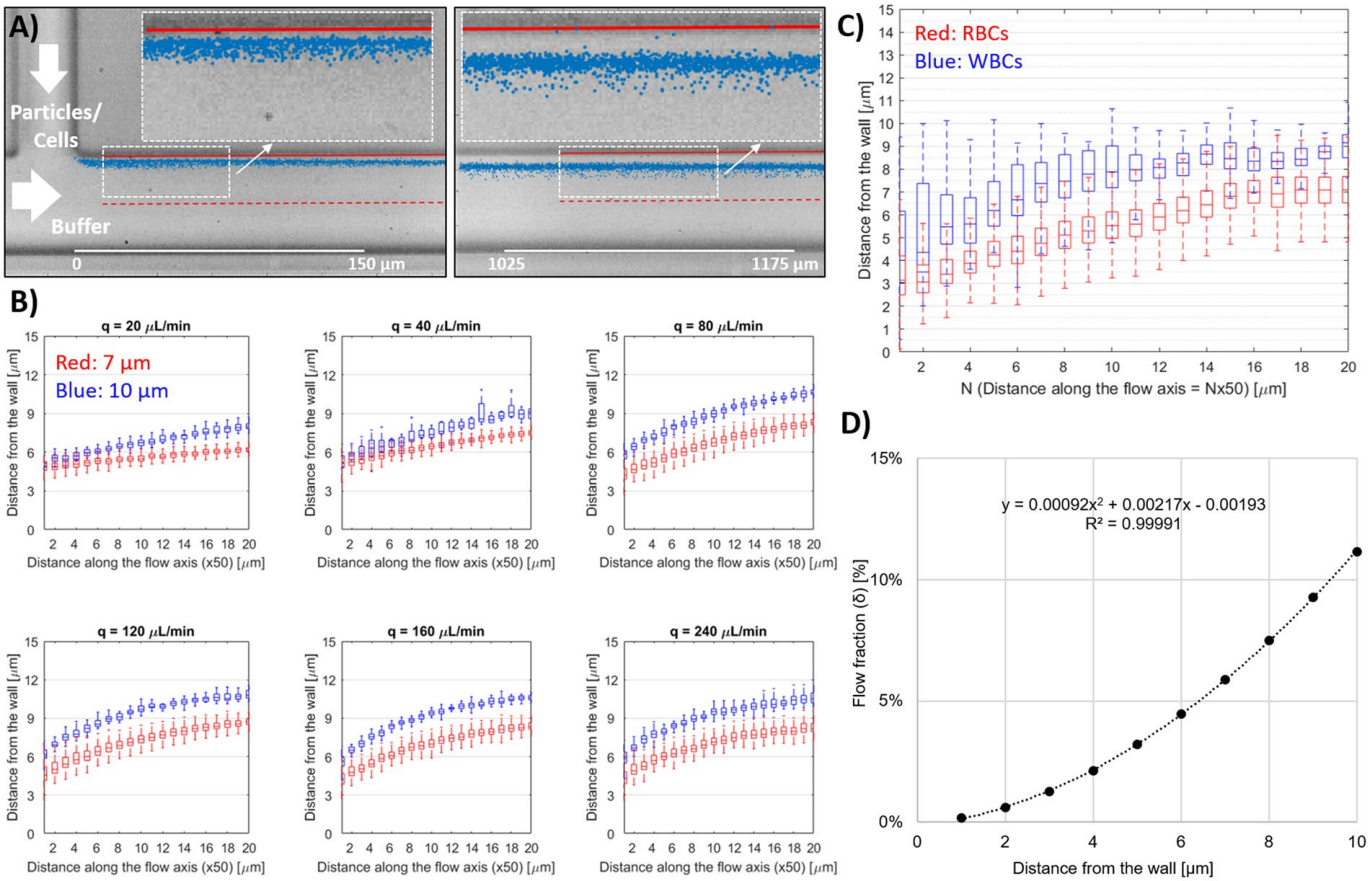
Characterization of wall-induced inertial lift and particle/cell migration (**A**) Analytical device used to characterize the migration behavior of the polystyrene particles or RBC/WBCs. Buffer flowrate is 40 times larger than the particle inlet flowrate, which ensures that the particles/cells are adjacent to the wall as they enter the lateral microchannel. Particles’ migration from the wall is tracked by a high-speed camera and their positions are determined via image processing (blue marks on the picture indicate center position of the particles/cells, red circles/lines indicate channel walls, marker borders and centerlines). (**B**) Wall migration results for 7 and 10 μm particles for a range of total flowrate per rows (*q* = 20–240 μL/min) in a 50 μm width/52 μm depth channel. (**C**) Wall migration results for RBCs and WBCs at *q* = 80 μL/min. (In the box plots, bottom and top edges of the box indicate the 25th and 75th percentiles and the whiskers indicate 99.3%). (**D**) Correlation between the distance of the particle from the wall and flow fraction (*δ*), based on a fully developed flow model in a 50 μm channel.

Characterization experiments were conducted to optimize the design parameters *L*, *U* and *τ*, to exploit the difference in non-equilibrium migration distances of particles/cells deflected away from a single island. These experiments were run initially with monodisperse particles to narrow down the parameter space, and then with blood cells for optimization. Particle diameters were selected as reference points for blood components: 10 μm is a nominal size for WBCs, whereas 7 μm is an upper size threshold for RBCs and a lower size threshold for WBCs. The single deflection chip (Fig. 2) consisted of two inlets (particles/cells and buffer), where buffer flow was used to pinch the particle flow to bring the particles/cells adjacent to the wall. Particle/cell migration trajectories along the channel were recorded with a high-speed camera (See Movies S1,S2), and then were processed via a custom image processing software running in MATLAB to locate particles/cells. In a typical processed image (Fig. 2A), the migration of the particles/cells away from the wall between where they were introduced into the channel (left) and downstream (right) could be clearly visualized.

A range of flowrate per rows (*q*) was investigated to modify *U*, and characterize its effect of on the particle migration trajectories. It was observed that both in 30 μm and 50 μm width channels (Figure S1 and Fig. 2B, respectively), a range of operating parameters (*q* and *L*) could be selected to achieve good separation between the migration trajectories of 7 and 10 μm particles. We opted to further investigate the wider (50 μm) channel design to alleviate the potential clogging problems of the chip during processing blood, as discussed later in the manuscript. At low flowrates (*q* ≤ 40 μL/min), a length of 300 μm was required to achieve significant separation between the migration trajectories (*i.e*. no overlap of the 25th/75th percentile boxes). However, at higher flowrates (*q* ≥ 80 μL/min), significant separation was achieved even within *L* ≤ 50 μm, and no overlap between particle trajectories was observed beyond *L* = 200 μm. Thus, for the particle size range of interest a minimum flowrate per row of 80 μL/min was required to achieve rapid inertial separation. Higher flowrates offered no significant advantage in terms of non-equilibrium separation, and thus were not investigated further as they would increase the driving pressure requirement of the device.

Based on these results, we investigated the migration of blood cells at *q* = 80 μL/min. As shown in Fig. 2C, RBCs and WBCs followed different migration trajectories, and WBCs migrated farther due to their larger size. The migration path of WBCs was found to lie in between trajectories of 7 and 10 μm particles, whereas the path of RBCs was below 7 μm particles. Compared to polystyrene particles, the migration path of the cells was more spread out, which was attributed to the higher variability of the physical characteristics (size, shape, deformability, *etc*.) of the cells. In particular, the variability of the RBCs’ migration paths could be explained by the discoid shape of the cells, and their orientations as they interact with the wall. WBCs are more heterogenous than RBCs, and the three major subpopulations of WBCs (Neutrophils, Lymphocytes, Monocytes) are all different in size: 10–12 μm, 7–8 μm, and larger than 15 μm respectively, which contributes to the observed variation in their migration. Furthermore, the nucleus of WBCs is more rigid than the more flexible cell membrane, which may reduce the effective diameter of the cell.

In order to determine the siphon fraction (*τ*), the flow fraction (*δ*) between the particle and the deflecting wall needs to be known. Since the relationship between *δ* and distance from the wall (*x*) is non-linear, a correlation was obtained from a 3D numerical simulation (via COMSOL) of a fully developed flow in the microchannel (Fig. 2D). Since *δ* and *x* are positively correlated, a higher *δ* implies that a particle migrated farther from the wall, and vice versa. The separation performance of the NISA design was investigated using a modified version of the single deflection chip (Figure S2) with a fixed island length (*L* = 200 μm) and two outlets (primary and siphon), and discussed in detail in the supporting information.

### Fractionation performance with whole blood

**Figure 3:**
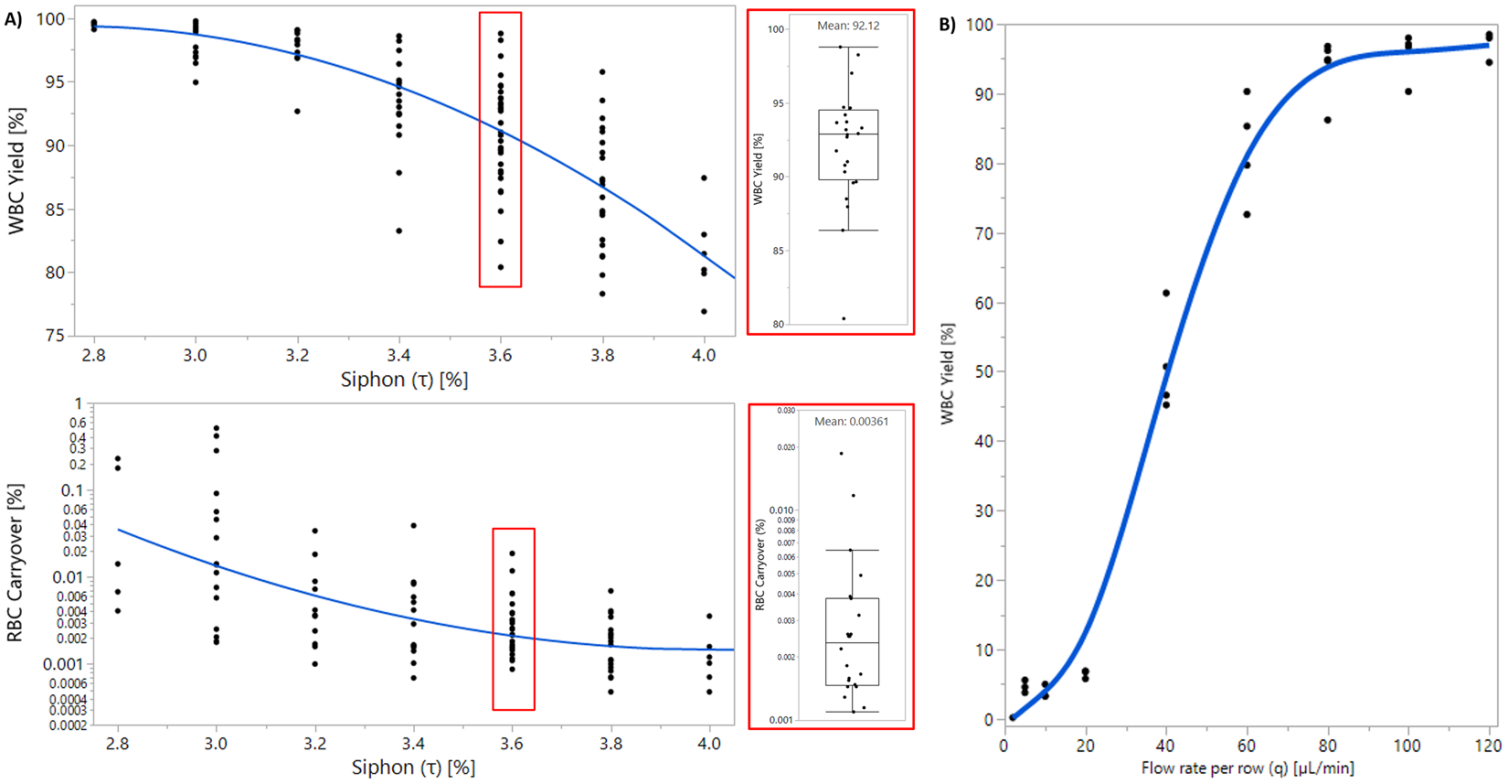
Analytical NISA device blood fractionation performance (**A**) Yield and purity of the WBC product at varying siphon percentages (*τ*) (Box panel shows detailed WBC yield and RBC carryover analysis of the optimized device with *τ* = 3.6% and *q* = 80 μL/min). (**B**) Yield of the WBCs with varying flowrates per row (*q*). Unlike a DLD, NISA mechanism does not work at low flowrates.

The main goal of whole blood fractionation was to obtain a high yield (*i.e*. minimal WBC loss to waste) and high purity (*i.e*. minimal RBC carryover to product) WBC product. Thus, the performance metrics were determined as: 1) WBC yield = (# of WBCs in product)/(# of WBCs in product + waste), and 2) RBC carryover = (# of RBCs in product)/(# of RBCs in product + waste). Initial blood fractionation experiments were conducted with an analytical device (See Table 1, Row 2). Siphon percentage (*τ*) was varied between 2.8% to 4% at a fixed flowrate per row (*q* = 80 μL/min). In these devices, the number of columns per array (*m*) changed between 25 to 42 depending on *τ*, to satisfy *m* × *τ* ≥ 100%. It was observed that the WBC yield fell as *τ* increased, from 99.3 ± 0.4% at *τ* = 2.8% down to 77.8 ± 10% at *τ* = 4.0% (Fig. 3A). At *τ* = 3.6%, the device achieved 92.1 ± 3.9% WBC yield, and 0.0036 ± 0.004% RBC carryover (Fig. 3A, boxed panel). The decrease in WBC yield with increasing *τ* is an expected result as more of the flow is siphoned, WBCs that have not migrated far enough are more likely to end up in the waste row. It was also determined that smaller WBCs (*e.g*. lymphocytes) constituted a larger fraction of this lost portion, as expected (See Figure S3, and SI for additional discussion). Because of the same mechanism, RBC carryover decreased from 0.056 ± 0.09% at *τ* = 2.8% down to 0.0013 ± 0.0009% at *τ* = 4.0%. Since both high WBC yield and small RBC carryover are desirable, a compromise was necessary. Hence, *τ* was fixed at 3.6% in the subsequent experiments. After selecting *τ*, the flowrate per row (*q*) was changed between 0 to 120 μL/min (Fig. 3B). It can be observed that at lower *q* values, WBC yields were very low (*i.e*. the device ceases to function) due to the positive correlation between the flow velocity and inertial lift force. Note that this is a primary difference between a DLD array and the current, inertial lift based design. Only for *q* ≥ 80 μL/min, a significant portion (>90%) of WBCs were retained in the product. While the main goal was to produce a WBC product, hemolysis analysis was also conducted to investigate any potential damage to RBCs during NISA processing. The results showed minimal damage to the RBCs (<0.5% increase in free plasma HGB per RBC), and no positive correlation was observed between the increased flowrate and hemolysis of the RBCs (See SI for additional details).

A fully-parallelized (104 devices) NISA device (see Figure S4 for the full experiment setup) was manufactured for the very large volume (400 mL) blood fractionation experiments. 104 parallel devices were fit on the top half of a 12-cm diameter COP disk, demonstrating the compact footprint and the scalability of the NISA architecture. Design and operation parameters of the fully-parallelized device were the same as the final configuration of the analytical device (*L* = 200 μm, *τ* = 3.6%, *q* = 80 μL/min). Initially, CFD simulations (using ANSYS Fluent 13) were used to verify that: (1) *τ* is correctly set by the device geometry, and (2) streamlines that make it to the product are at the desired distance from the island wall so that separation between WBCs and RBCs can be achieved (Figure S5a). CFD simulations (calculated over a full array of islands) verified that *τ* varies minimally between islands of the same row, and mean values of τ for row 1 (where the separation primarily occurs) and row 2 were: τ_row1 to waste_ = 0.035 ± 0.003 and τ_row2 to row1_ = 0.034 ± 0.005, respectively. τ_product to row2_ does not contribute to the performance of the device, as long as it is sufficiently small (<< 3.6%) to avoid siphoning of the WBCs. Streamlines which are generated at the end of an island illustrate the flow and verify that the proposed mechanism for separation, that of a size-dependent inertial lift, is primarily responsible for separation in the NISA device. Specifically, it can be observed that if the cells follow streamlines when they reach to the end of an island, according to their center position, WBCs will make it to the next island and RBCs will get siphoned based on their migration distance at *L* = 200 μm and *q* = 80 μL/min (Fig. 2C).

In order to verify that the cells follow streamlines faithfully, we calculated the relevant Stokes numbers (*St*) for the separate cases we investigated as: *St* = *ρ*_*p*_ *a* ^*2*^ *U*_*m*_ */18μL*_*c*_, where *ρ*_*p*_ is the particle density, *μ* is the viscosity of the carrier fluid, *U*_*m*_ is the mean per-channel flow velocity and *L*_*c*_ is the characteristic length, which was selected as 50 μm (equal to the width of the channel, distance between the islands, and nearly identical with channel height). For the final per-channel flowrate of the NISA device (*q* = 80 μL/min), we calculated *St* = 0.057 and *St* = 0.028 for the 10 μm and 7 μm particles in the buffer solution. For the same channel flowrate, we evaluated *St* = 0.029 and *St* = 0.014 for the WBCs and RBCs in 1:1 diluted whole blood. Note that even though we used the same particle dimensions for both calculations, the WBCs and RBCs in diluted blood have a lower Stokes number because of the higher viscosity of the diluted blood compared to the buffer solution. Since the 10 μm particles in the buffer solution have the highest *St*, we further analyzed that case using a particle tracing CFD model (ANSYS Fluent 13, discrete phase model) to visualize how faithfully those particles followed the streamlines. The CFD simulation (Figure S5b) showed that even in the higher *St* condition (larger particles in the buffer solution), the separation mechanism was not significantly affected by the particle inertia, such that particle path lines deviated minimally from the streamlines (inter-streamline distance was 0.5 μm at the end of the island). Because the estimated Stokes number for 10 μm particles in buffer is << 1 and the particle tracing simulation results indicated minimal deviation between streamlines and 10 μm diameter particle path lines, we conclude that cells in blood (which have an even lower *St*) follow streamlines sufficiently faithfully and the separation mechanism is not significantly affected.

**Figure 4:**
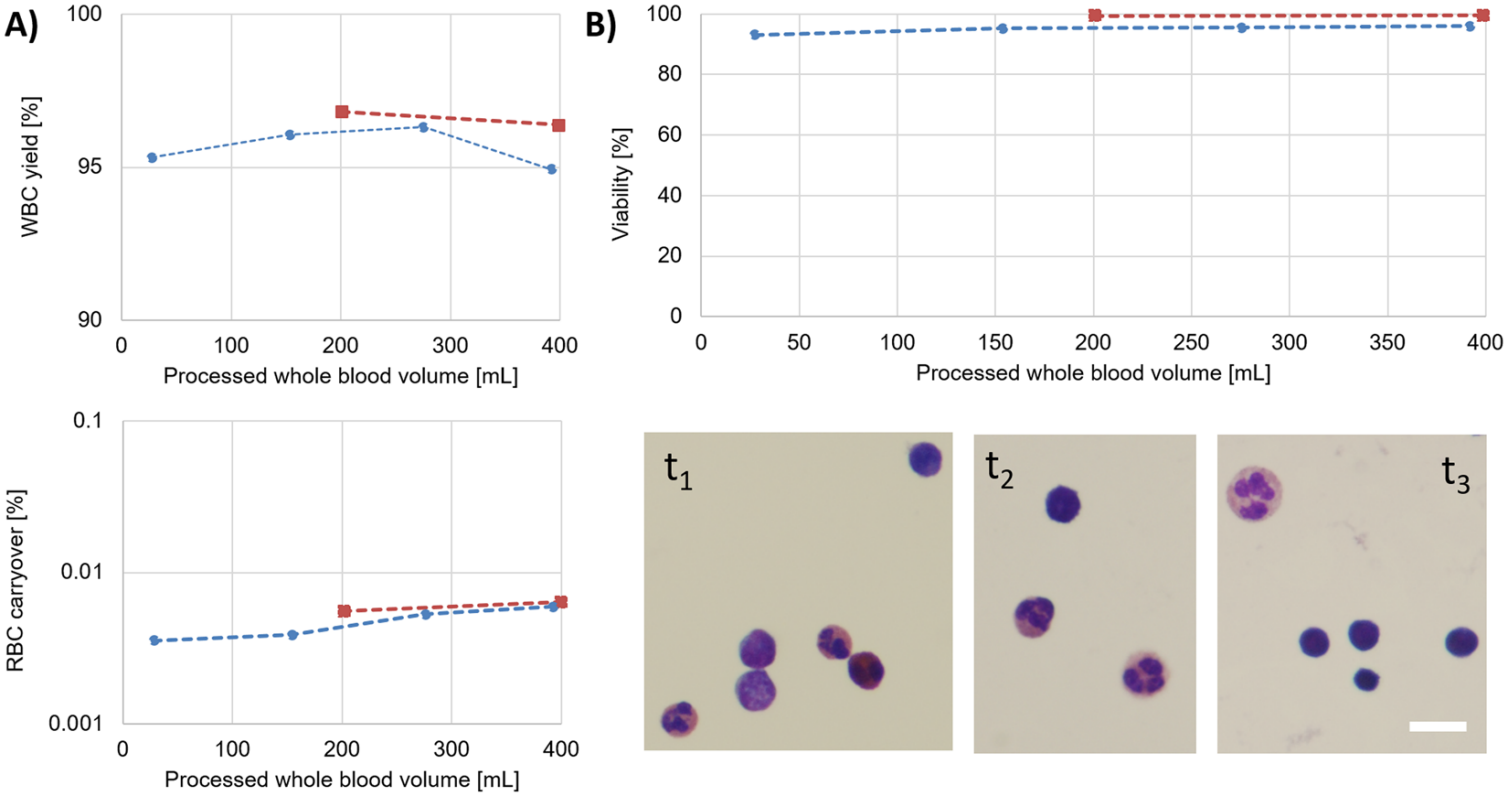
Fully-parallelized NISA device performance processing very large volume (~400 mL) of blood: (**A**) WBC yield and RBC carryover analysis of the parallelized NISA device from two separate runs (**B**) WBC product analysis at multiple time intervals using: Sytox green viability (top), and Wright-Giemsa staining (bottom). Time points for the Wright-Giemsa staining are selected based on the processed blood volume as: t_1_ = 153 mL, t_2_ = 275 mL and t_3_ = 392 mL (Scale-bar is 10 μm).

The performance of the device was evaluated by collecting samples continuously, until a pre-set time point. The fully-parallelized device was able to process 400 mL of whole blood within three hours, with 96.6 ± 0.3% and 95.7 ± 0.6% WBC yield and 0.0059 ± 0.0006% and 0.0047 ± 0.0010% RBC carryover (for the first and second run respectively), averaged over the collected fractions at different time points (Fig. 4A). Results were well within the range of performance metrics obtained with small volume blood processing (Compare to Fig. 3), which verified that the performance of the device was consistent over three hours of operation. The fractionated WBCs were also evaluated for viability (by membrane integrity using Sytox Green stain) and cell morphology (Wright-Giemsa stain). For the two runs, cell viability was measured as 99.6 ± 0.6% and 94.9 ± 1.3%, respectively (Fig. 4B). All subpopulations of WBCs (neutrophils, lymphocytes and monocytes) were morphologically normal, with no observable disruption to the cell structure (Fig. 4B). Therefore, we concluded that the WBCs were not negatively affected by going through the device.

Large volume experiment results showed that the NISA device was able to continuously fractionate blood for three hours without clogging, which is a major problem with blood processing microfluidic chips^25–27^. Clogging is due to aggregation of blood components, caused by shear stress induced activation of platelets and von Willebrand factor (vWF) fibers^28–30^. Therefore, NISA mechanism offers a major advantage by requiring minimal contact of the blood components with the device features to reduce this effect. However, high shear stresses are still unavoidable in a high-throughput, large-volume microfluidic blood processing device, thus our design criterion was to promote easy and quick removal of any aggregate from the chip before causing performance issues or device failure. In order to achieve this, NISA device was designed to incorporate: 1) Large channel width (50 μm) compared to cell size, and 2) Small residence time (≪1 second). The first point can be highlighted by comparison with two DLD architectures previously reported by our research group^14^. These devices had 99.7% and 59% nucleated cell yield (mostly WBCs), when the distance between the posts was 20 μm and 32 μm, respectively. In that design, the substantial decrease in the nucleated cell yield was unavoidable when the gap distance was increased, due to the DLD’s separation mechanism which relies on contact between cells and posts. However, in this study, we were able to use a 50 μm channel width and still attain >95% WBC yield. It should be noted that increasing the channel height (>52 μm) is also a feasible option to facilitate blood processing at even higher throughputs using the NISA mechanism. However, depending on the manufacturing method of the large-scale device (*e.g*. injection molding), slight wall angles may be introduced; the effects of which would get more pronounced with higher channels due to geometry. Thus, we did not investigate taller channels in this study. Addressing the second point, the small residence time of the blood components in the NISA device is enabled by the positive correlation between WBC yield and flowrate (See Fig. 3B). As a matter of fact, high flowrates are required for the NISA mechanism to work, as compared to a DLD. In contrast, in a DLD architecture, high flowrates have been shown to deform the cells and change their effective size^31^, which can reduce the separation efficiency of the device.

## Conclusion

In this study, we describe a precise (isolation of targeted cells with high yield and purity), gentle (minimal manipulation), and high-throughput microfluidic device for blood fractionation, capable of processing a large volume (400 mL) of whole blood at a rate of 3 mL/min, or approximately 300 million cells/second. Inertial microfluidics, which form the basis of the presented NISA device, enable precise manipulation of cells and is used in a wide array of cell focusing applications^32^. The NISA device, instead of focusing the cells, utilizes the wall-induced inertial lift force and exploits the difference in the size-based migration trajectories of blood cells. Using this mechanism, the larger cells (WBCs) are separated from the smaller ones (RBCs) before reaching focus positions, and a high yield and purity buffy coat is produced. The throughput of the device is comparable to bench-top techniques, making it a feasible alternative to existing technologies for the production of blood products. In addition, a high throughput cell sorting device allows for potential new applications, including rare cell enrichment, intraoperative blood salvage, blood purification, and banked blood cleansing^27^. The size cut-off of the NISA device can be easily tuned to permit such applications, without compromising throughput.

## Methods

### Particle/cell migration and separation characterization

PDMS devices (single deflection chips) were fabricated using standard soft lithography techniques^33^. For the migration and separation characterization experiments, width of the main channel was 30 μm or 50 μm, and the channel height was 52 μm. In separation characterization devices, siphon percentage (*τ*) of the devices were adjusted by adding microfluidic resistance (0.0099” ID Tygon Tubing, Cole Parmer) to the siphon or primary outlet. Monosized polystyrene particles (NIST, Polysciences Inc.) were suspended in PBS solution and the solution was density matched with respect to the corresponding particle density using Optiprep (Sigma Aldrich). Fresh human whole blood from healthy volunteers aged 18 years and older was commercially sourced from Research Blood Components (Boston, MA) or obtained with informed consent of healthy donors according to an institutional review board approved protocol at the Massachusetts General Hospital. In both cases peripheral blood was drawn into acid citrate dextrose (ACD) tubes (Vacutainer, Becton Dickinson). For RBC experiments, whole blood was diluted 1000x to bring the RBC concentration to approximately 5 × 10^6^ cells/mL and used without further processing. For WBC experiments, a previously designed DLD-based microfluidic chip^14^ was used (without the magnetic sorting) to obtain buffy coat from whole blood. Both RBC and WBC cell suspensions were also density matched as previously described. Syringe pumps (Harvard Apparatus) were used to deliver the solutions to the microchannels. The ratio of particle/cell flow to the buffer flow was kept constant at 1:40 [v/v] for all the tested flowrates. A high-speed camera (Phantom 4.2, Vision Research Inc.) taking images at 4,400 frames/second was used to record the migration of the particles and cells.

### Blood fractionation

Fully-parallelized (104 devices) NISA device, and quadrupled (4 devices) analytical devices with varying siphon percentages (*τ*) from 2.8 to 4.0% were manufactured of cyclic olefin polymer (COP) by Stratec Biomedical AG (Germany). The device designs included a microfluidic filter on the inlets (blood and buffer) which had a 30 μm gap distance, to remove any initial aggregate or debris. For the fully-parallelized device, 26 inlet holes for sample and 52 inlet holes for the buffer were addressed by millimeter-scale channels embedded within the interface layer (visible in Figure S4, #3). The result is that each sample hole feeds blood to four arrays, by channels that never drop below the 50 μm minimum already present within the debulking arrays, and each buffer hole feeds buffer to two arrays. This design ensures that the shear stress experienced by blood cells in their trip to the array is not higher than within the debulking arrays. Blood was obtained as described in the previous section. In small volume experiments, 1.5 mL of whole blood was diluted 1:1 with 1% w/v F-127 in PBS and loaded into a 3 mL BD syringe. Buffer solution (1% w/v F-127 in PBS) was loaded into a 10 mL BD syringe. F-127 was added to the buffer to aid priming of the chip by reducing the surface tension (Pluronic F-127, Sigma Aldrich). F-127 also helps to prevent blood components from sticking to channel walls during the run. Constant flowrate was maintained by syringe pumps (Harvard Apparatus), where the sample injection fraction of 1:1 diluted whole blood was maintained at 18% [v/v] of the total flowrate. The NISA device was primed for 1 minute with the buffer before introducing blood into the system. The WBC product and NISA waste (containing platelets, red blood cells, and plasma) were collected into separate containers (polypropylene and polyethylene, respectively) to prevent cell-sticking. Blood cells in the product were counted using a Sysmex XP-300 hematology analyzer, a Neubauer hemocytometer, or a Nageotte bright-line hemocytometer chamber. Mean cell hemoglobin (MCH) of the input sample was measured using a Sysmex XP-300 hematology analyzer. To determine the free plasma hemoglobin of the input sample and the waste output, samples were centrifuged at 200 rcf for 20 minutes to pellet the RBCs. Then the hemoglobin (HGB) content of the plasma was measured using a hemoglobin assay kit (MAK115, Sigma-Aldrich, MO). For the very large volume (~400 mL) experiments, WBC product and waste output fractions were collected and characterized similar to the small volume experiments. In addition, the viability and morphology of the WBCs in the product were also evaluated. Cell viability in the product was measured by staining samples with Sytox Green cell-permeant nucleic acid stain and using flow cytrometry to determine the concentration of fluorescing dead cells. Cells in the product were also stained with Wright-Giemsa to visualize potential aberrations in cell morphology.

## Electronic supplementary material

Supplementary information

High-speed video of a 7 μm particle migrating away from the wall in a single deflection chip at channel flowrate of q = 80 μL/min

High-speed video of a 10 μm particle migrating away from the wall in a single deflection chip at channel flowrate of q = 80 μL/min

## References

[1] Ganzel C, Becker J, Mintz PD, Lazarus HM, Rowe JM (2012). Hyperleukocytosis, leukostasis and leukapheresis: Practice management. Blood Rev..

[2] Meyer TPH (2005). Filter Buffy Coats (FBC): A source of peripheral blood leukocytes recovered from leukocyte depletion filters. J. Immunol. Methods.

[3] Riethdorf S (2007). Detection of circulating tumor cells in peripheral blood of patients with metastatic breast cancer: A validation study of the CellSearch system. Clin. Cancer Res..

[4] Bianchi DW (1993). Erythroid-specific antibodies enhance detection of fetal nucleated erythrocytes in maternal blood. Prenat Diagn.

[5] Pantel K, Brakenhoff RH, Brandt B (2008). Detection, clinical relevance and specific biological properties of disseminating tumour cells. Nat. Rev. Cancer.

[6] Reátegui E (2015). Tunable nanostructured coating for the capture and selective release of viable circulating tumor cells. Adv. Mater..

[7] Stott SL (2010). Isolation of circulating tumor cells using a microvortex-generating herringbone-chip. Proc. Natl. Acad. Sci.

[8] Nagrath S (2007). Isolation of rare circulating tumour cells in cancer patients by microchip technology. Nature.

[9] Hou HW (2011). Microfluidic devices for blood fractionation. Micromachines.

[10] Toner M, Irimia D (2005). Blood-on-a-Chip. Annu. Rev. Biomed. Eng..

[11] American Red Cross. Blood facts and statistics. Available at: http://www.redcrossblood.org/learn-about-blood/blood-facts-and-statistics. (Accessed: 9th January 2017).

[12] Huang Lotien, Richard SJC (2004). Continuous Particle Separation Through Deterministic Lateral Displacement. Science (80−.).

[13] Huang R (2006). A microfluidics approach for the isolation of nucleated red blood cells (NRBCs) from the peripheral blood of pregnant women. Prenat. Diagn..

[14] Ozkumur E (2013). Inertial Focusing for Tumor Antigen-Dependent and -Independent Sorting of Rare Circulating Tumor Cells. Sci. Transl. Med..

[15] Loutherback K (2012). Deterministic separation of cancer cells from blood at 10 mL/min Deterministic separation of cancer cells from blood at 10 mL/min. AIP Adv.

[16] D’Silva J, Austin RH, Sturm JC (2015). Inhibition of clot formation in deterministic lateral displacement arrays for processing large volumes of blood for rare cell capture. Lab Chip.

[17] McGrath J, Jimenez M, Bridle H (2014). Deterministic lateral displacement for particle separation: a review. Lab Chip.

[18] Hur SC, Tse HTK, Di Carlo D (2010). Sheathless inertial cell ordering for extreme throughput flow cytometry. Lab Chip.

[19] Hou HW (2013). Isolation and retrieval of circulating tumor cells using centrifugal forces. Sci. Rep.

[20] Warkiani ME (2016). Ultra-fast, label-free isolation of circulating tumor cells from blood using spiral microfluidics. Nat. Protoc..

[21] Gossett DR (2012). Inertial manipulation and transfer of microparticles across laminar fluid streams. Small.

[22] Carlo DDi (2009). Inertial microfluidics. Lab Chip.

[23] Di Carlo D, Edd JF, Humphry KJ, Stone HA, Toner M (2009). Particle segregation and dynamics in confined flows. Phys. Rev. Lett..

[24] Martel JM (2015). Continuous Flow Microfluidic Bioparticle Concentrator. Sci. Rep.

[25] Chung J (2012). Microfluidic cell sorter (μFCS) for on-chip capture and analysis of single cells. Adv. Healthc. Mater.

[26] Loutherback K (2010). Improved performance of deterministic lateral displacement arrays with triangular posts. Microfluid. Nanofluidics.

[27] Warkiani ME, Wu L, Tay AKP, Han J (2015). Large-Volume Microfluidic Cell Sorting for Biomedical Applications. Annu. Rev. Biomed. Eng..

[28] Schneider SW (2007). Shear-induced unfolding triggers adhesion of von Willebrand factor fibers. Proc. Natl. Acad. Sci. USA..

[29] Ramstack JM, Zuckerman L, Mockros LF (1979). Shear-induced activation of platelets. J. Biomech..

[30] Holme PA (1997). Shear-Induced Platelet Activation and Platelet Microparticle Formation at Blood Flow Conditions as in Arteries With a Severe Stenosis. Arterioscler. Thromb. Vasc. Biol..

[31] Beech JP, Holm SH, Adolfsson K, Tegenfeldt JO (2012). Sorting cells by size, shape and deformability. Lab Chip.

[32] Martel JM, Toner M (2014). Inertial focusing in microfluidics. Annu. Rev. Biomed. Eng..

[33] Duffy DC, McDonald JC, Schueller OJA, Whitesides GM (1998). Rapid prototyping of microfluidic systems in poly(dimethylsiloxane). Anal. Chem..

