# Supplementary material for: Non-equilibrium Inertial Separation Array for High-throughput, Large-volume Blood Fractionation
Source: Sci Rep. 2017 Aug 30;7:9915. doi: 10.1038/s41598-017-10295-0 (PMC5577162; doi:10.1038/s41598-017-10295-0)
Supplement: Supplementary file 1 — Supplementary information [file 41598_2017_10295_MOESM1_ESM.doc]

**Non-equilibrium Inertial Separation Array for High-throughput, Large-volume Blood Fractionation**

Baris R. Mutlu ^1,†^, Kyle C. Smith ^4,†^, Jon F. Edd ^2^, Priyanka Nadar ^4^, Mcolisi Dlamini ^1^, Ravi Kapur ^1,2,4^, Mehmet Toner ^1,3,*^

**Supporting Information**

**Physics of NISA mechanism**

Any particle of non-negligible size suspended in a carrier fluid which is flowing in a straight microchannel (*i.e.* no Dean flow) is subjected to two inertial lift forces (*F_L_*). These forces are: (I) Wall lift force, which arises from the interaction of the particle with the channel wall, (II) Shear gradient force, which is due to the curved velocity profile of the flow ^1^. The combination of these forces is often represented by a single equation: *F_L_ = f_L_ρU^2^a^4^/H^2^*, where *f_L_* is a dimensionless lift coefficient, *ρ* is the density of the carrier fluid, *U* is the mean flow velocity in the channel, *a* is the particle diameter and *H* is the channel height. It is readily apparent that the lift force is strongly correlated with the size of the particle (*a^4^*). Furthermore, Di Carlo *et al.* have shown that *f_L_* scales with *(a/H)^2^* near the channel wall, resulting in a stronger correlation between the lift force and the particle size (*F_L_* ~ *a^6^*) ^2,3^. Thus, different size particles have different migration trajectories to their inertially focused positions. The critical design and operation parameters of the NISA device include: island length (*L*), flow velocity (*U*) and siphon fraction (*τ*). *L* and *U* directly affect the duration that the particles are subjected to the wall lift force, whereas *U* is also linearly correlated with *F_L_*. Thus, in combination, these two parameters dictate the migration path of the particles/cells away from the wall. Siphon fraction (*τ*) is defined as the volumetric fraction of the fluid that is separated from the main flow before particles reach equilibrium. This separation is achieved by adjusting the downstream hydraulic flow resistances (*e.g.* decreasing the channel width to increase hydraulic resistance). The magnitude of *τ* dictates whether a particle is going to stay in the main flow, or get siphoned as “waste”, based on its lateral position in the channel. Thus, it is selected based on the migration distance of the particle of interest at a given *L* and *U*, and the corresponding fraction of the flow between the particle and the channel wall.

**Characterization of particle separation**

The separation performance of the NISA design was investigated using a modified version of the single deflection chip with a fixed island length (*L* = 200 μm) and two outlets (primary and siphon) (Figure S2A). A mixed population of polystyrene particles was used to ensure monodisperse size and comparable population density of the particles. This chip was designed such that it siphoned a fixed fraction (*τ*) of the flow to the siphon outlet. The design principle is that 7 and 10 μm particles are separated only if *τ* satisfied *δ*_7_ < *τ* < *δ*_10_ for the given island length. The number of particles in the outlet volumes were counted, and plotted as a function of channel flowrate (*q*) and *τ* (Figure S2B, top). At high *τ* values, both 7 and 10 μm particles were siphoned as the migration of the particles was not sufficient to escape from the siphoning flow (*δ*_7_ < *δ*_10_ < *τ*). However, as *τ* was decreased, the 10 μm particles started appearing in the primary outlet, followed by the 7 μm particles. The *τ* at which the particles started appearing in the primary outlet increased as the flowrate increased, due to the increased migration of the particles because of the larger wall lift force. The difference between the migration profile of the 10 μm particles and the 7 μm particles (Figure S2B, top) was used to quantify the separation performance. Using this metric (Figure S2B, bottom), 0% separation indicated no separation (all 7 and 10 μm particles in the same outlet) whereas 100% separation indicated perfect separation (all 7 μm particles in siphon and all 10 μm particles in the primary outlet). The separation performance increased with flowrate up to 120 μL/min, and the point of maximum separation rose as *q* increased.

**Analysis of WBC subpopulation yield based on siphon percentage (*τ*)**

Smaller WBCs (*e.g.* lymphocytes) are expected to constitute a larger fraction of this lost portion, due to the size dependence of the inertial lift force. This hypothesis was verified by further analysis of the WBC subpopulations (lymphocytes, neutrophils and monocytes) in the blood sample before and after fractionation. We calculated the relative yield of a specific WBC subpopulation by dividing the number of cells in the WBC product by the number of cells in the initial sample to (Figure S3). It was observed that as *τ* was increased, the yield of lymphocytes decreased, while the yield of the other WBCs (neutrophils and monocytes) stayed relatively constant. Thus, the overall decrease in the WBC yield with increasing *τ* was attributed primarily to the loss of lymphocytes, as expected.

**Hemolysis analysis**

Hemolysis analysis was conducted to evaluate any potential damage to RBCs after NISA processing by measuring the free hemoglobin (HGB) concentration in the plasma (cell free portion of the waste output). Plasma HGB concentration was then normalized based on the RBC count of the waste output to obtain the HGB mass in the plasma per RBC (here named MxCH, similar to mean cell hemoglobin or MCH). For the 3 different flowrates tested, MxCH was measured as: 0.11 ± 0.05, 0.10 ± 0.07 and 0.10 ± 0.08 pg/cell, and MxCH/MCH was calculated as: 0.42 ± 0.17%, 0.39 ± 0.26% and 0.39 ± 0.28% for q = 40, 80 and 160 μL/min respectively. The results show that less than 0.5% of RBC hemoglobin was found in the plasma, suggesting minimal damage to the RBCs during NISA processing. This is further supported by the fact that no positive correlation was observed between the increased flowrate (*i.e.* increased shear stress) and hemolysis of the RBCs.

**References**

1. Carlo, D. Di. Inertial microfluidics. *Lab Chip* **9,** 3038–3046 (2009).

2. Di Carlo, D., Edd, J. F., Humphry, K. J., Stone, H. A. & Toner, M. Particle segregation and dynamics in confined flows. *Phys. Rev. Lett.* **102,** 1–4 (2009).

3. Gossett, D. R. *et al.* Inertial manipulation and transfer of microparticles across laminar fluid streams. *Small* **8,** 2757–2764 (2012).

Figure S1


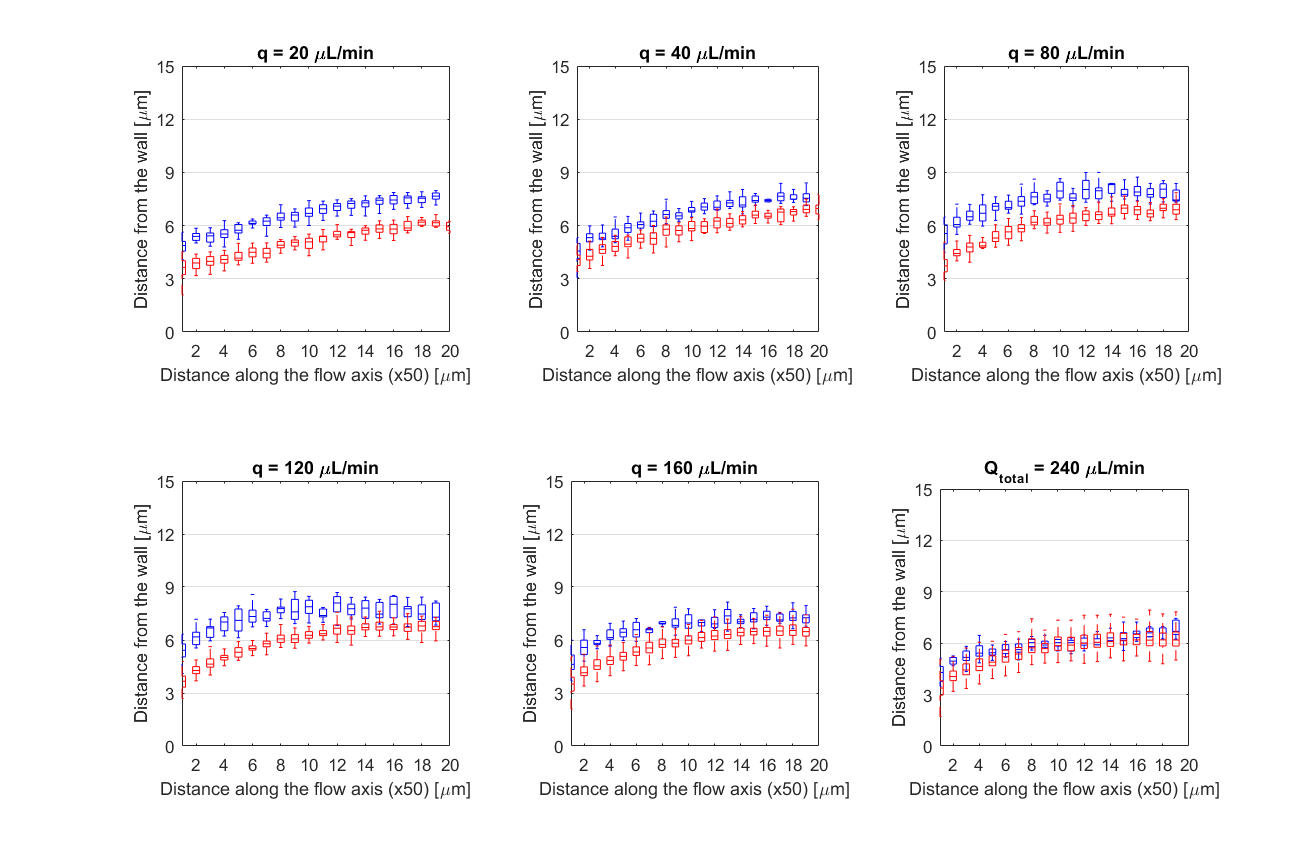


**Characterization of wall-induced inertial lift and particle migration:** Wall migration results for 7 and 10 μm particles for a range of total channel flowrates (*q* = 20-240 μL/min) in a 30 μm width / 52 μm depth channel.

Figure S2


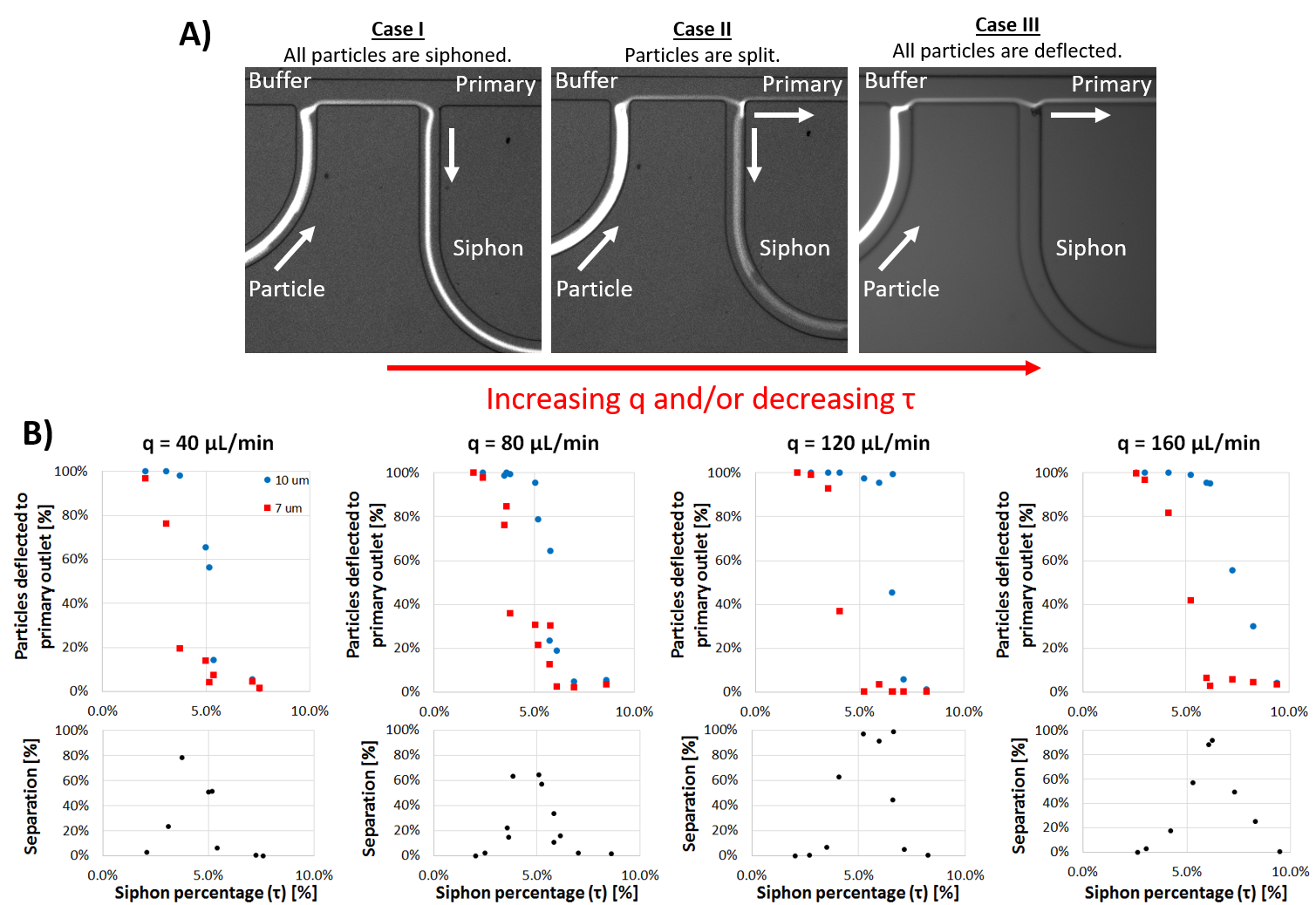


**Non-equilibrium separation of particles using a single island: A)** Streak images of a single island (200 μm length) with fluorescent 10 μm particles showing that by changing the siphon fraction (*τ*) or increasing the flowrate (*q*), particles can be alternately directed to the siphon or primary outlet. **B)** Particle separation experiment results illustrate that particles of different sizes can be separated using the wall lift force.

Figure S3


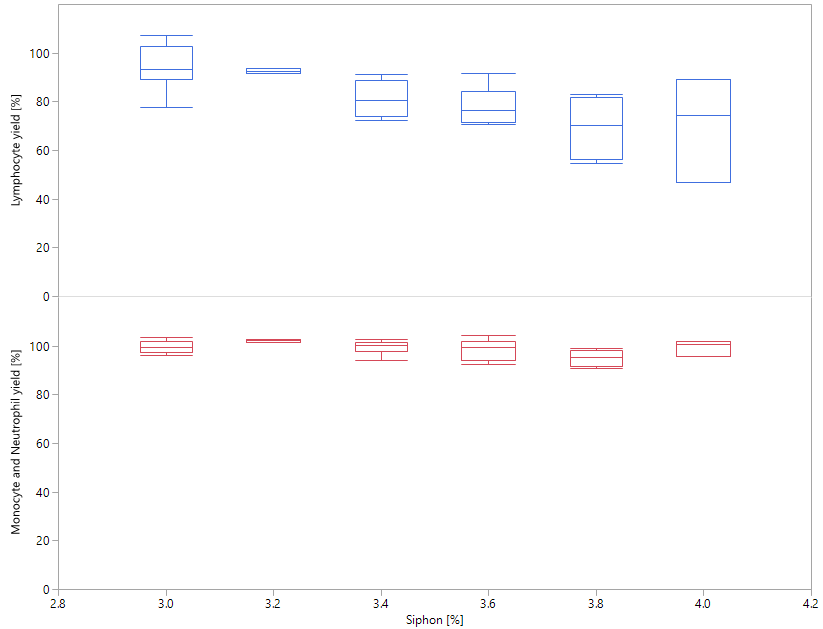


**NISA device fractionation performance of different WBC subpopulations:** Yield of the WBC subpopulations at varying siphon percentages (*τ*): Small WBCs (Lymphocytes) and Large WBCs (Monocytes and Neutrophils) shown separately.

Figure S4


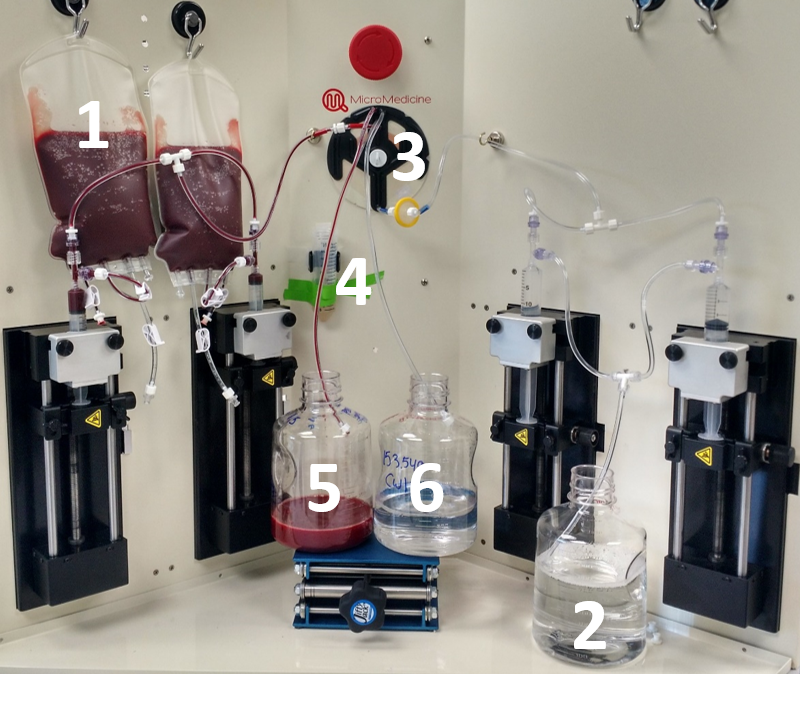


**NISA device processing a large volume (~400 mL) of blood:** 1) Whole blood diluted 1:1 with buffer, 2) Buffer solution, 3) Chip containing the fully-parallelized NISA device and the concentrator. The black layer on top of the device is used to distribute the sample and the buffer solution to the different arrays, and is designed to ensure that the shear stress experienced by the cells in this layer is lower than the NISA device. 4) White blood cell (WBC) product collection, 5) NISA waste containing red blood cells (RBCs) and platelets, 6) Concentrator waste containing extracted buffer solution.

Figure S5


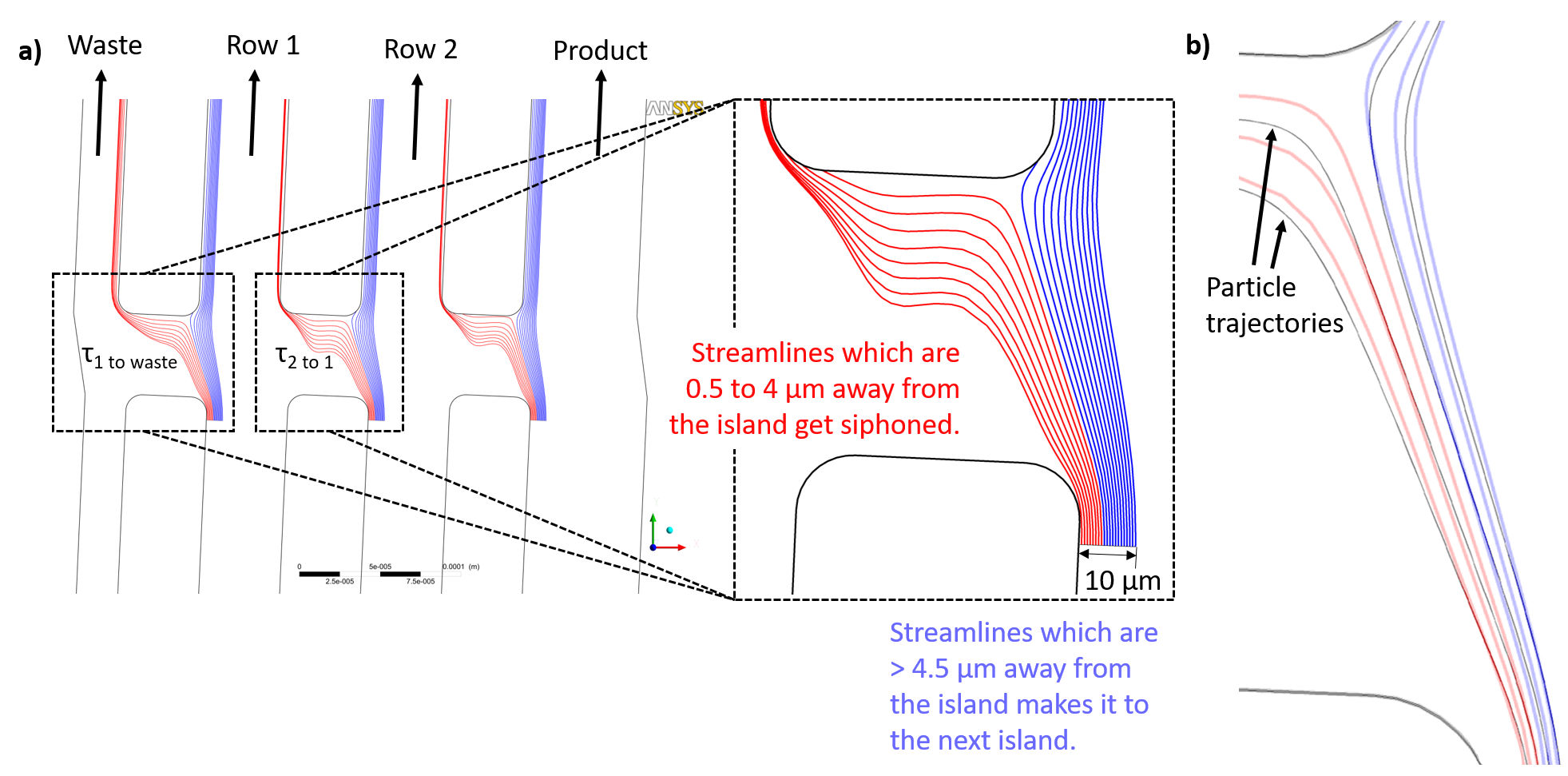


**CFD simulation of the 3D flow in the optimized NISA device (*q* = 80 μL/min): a)** Streamlines which are generated at the end of an island illustrate the flow, and are used to evaluate the portion of the flow that gets siphoned (τ), **b)** Particle trajectories overlayed with streamlines, showing that the separation mechanism is not significantly affected by the inertia of the particles at *St* = 0.057 (Note that the trajectories do not account for particle contact with the walls.)

Movie S1

High-speed video of a 7 μm particle migrating away from the wall in a single deflection chip at channel flowrate of *q* = 80 μL/min

Movie S2

High-speed video of a 10 μm particle migrating away from the wall in a single deflection chip at channel flowrate of *q* = 80 μL/min
